# Cognitive Load and Dual-Task Performance in Individuals with and without Forward Head Posture

**DOI:** 10.3390/jcm13164653

**Published:** 2024-08-08

**Authors:** Shorouk Abu-Ghosh, Ibrahim M. Moustafa, Amal Ahbouch, Paul A. Oakley, Deed E. Harrison

**Affiliations:** 1 Department of Physiotherapy, College of Health Sciences, University of Sharjah, Sharjah 27272, United Arab Emiratesiabuamr@sharjah.ac.ae (I.M.M.); aahbouch@sharjah.ac.ae (A.A.); 2 Neuromusculoskeletal Rehabilitation Research Group, RIMHS—Research Institute of Medical and Health Sciences, University of Sharjah, Sharjah 27272, United Arab Emirates; 3 Faculty of Physical Therapy, Cairo University, Giza 12613, Egypt; 4 Independent Researcher, Newmarket, ON L3Y 8Y8, Canada; docoakley.icc@gmail.com; 5 Kinesiology and Health Science, York University, Toronto, ON M3J 1P3, Canada; 6 Chiropractic Biophysics NonProfit, Inc., Eagle, ID 83616, USA

**Keywords:** craniovertebral angle, forward head posture, cognition, cognitive function, gait, dual task

## Abstract

**Background:** Recent studies have found forward head posture (FHP) is associated with altered physiology. There is a lack of research into whether FHP is associated with altered gait parameters when cognitively challenged. Our hypothesis is that individuals with FHP and those without will demonstrate different responses when undergoing dual-task assessment. **Methods:** Forty-five asymptomatic participants with FHP, defined as a craniovertebral angle (CVA) < 50°, were matched to forty-five participants with normal head posture (NHP) with a CVA > 55°. Participants walked along a 10 m platform under a control condition (no cognitive load) while an optical motion-capture system assessed gait kinematics. Secondly, participants were assessed under a dual-task cognitive load condition to identify the impact on gait kinematics. **Results:** Under the single-task condition, there were no significant differences for any gait parameter. In the dual-task condition, 12/13 gait parameters were significantly altered for the FHP vs. NHP group (*p* < 0.01). A calculation of the dual-task cost (DTC) percentage showed significant increases in all gait parameters in participants with FHP (*p* < 0.02). Correlations between the CVA and gait parameters were not significant for the single-task condition, but all gait parameters were correlated to CVA for the dual-task condition (*p* < 0.01). The correlation between CVA and DTC for all gait variables was significant (*p* < 0.04). **Conclusions:** This study demonstrates that FHP significantly increases the cognitive cost during walking, highlighting the importance of proper postural alignment for maintaining cognitive function under a dual-task condition.

## 1. Introduction

The debate surrounding forward head posture revolves around the question of whether it should be considered a normal variant or an abnormal condition that requires correction [1]. Forward head posture (FHP), where the head protrudes forward relative to the shoulders [2], is a common phenomenon in today’s digital age of increased screen time and sedentary lifestyles [3].

While pain can be a significant indicator of postural disorders [4], solely relying on pain to signify the presence or absence of FHP can be misleading. Not everyone with FHP experiences immediate pain or discomfort; some individuals may develop compensatory mechanisms to cope with their posture [5], which can temporarily mask pain but exacerbate problems in the long run. The situation of a lack of current pain might lead individuals to overlook the importance of diligently working to attain a more correct posture. It is essential to recognize that a comprehensive approach that goes beyond pain assessment ensures a more nuanced understanding of FHP and helps to uncover the underlying causes and potential risk factors associated with FHP.

One of the most common measurement methods for the quantification of FHP is the craniovertebral angle (CVA) [6,7]. A larger CVA value indicates a more desirable alignment of the head and neck, while a smaller angle indicates a more severe degree of FHP [5,6,7,8,9]. Participants with a CVA of less than 50° are generally classified as abnormal with significant FHP and have a higher frequency of cervical spine disorders such as neck pain and headaches [5,6,7,8,9]. In contrast, people with a CVA greater than 55° are classified as normal, with very little to no significant FHP and these individuals tend to be relatively symptom-free as they are more frequently found in asymptomatic control populations in case—control investigations [5,6,7,8,9]. In regard to asymptomatic individuals with FHP, previous research has presented an analysis of specific physiological parameters related to the presence of FHP [10,11,12,13,14]. The correlation between FHP in asymptomatic individuals and its impact on cervical spine nerve root evoked potentials [10], autonomic nervous system activity and sensorimotor control [11], central sensory processing and sensorimotor integration [12,13], athletic performance [13], and cardiopulmonary function [14] have been investigated. Building upon this intriguing groundwork, our current emphasis is on the captivating exploration of the relationship between posture displacement (termed subluxation or altered alignment [1]) and cognitive function. This topic has emerged as a compelling avenue for further research and understanding with only sparse information available on how FHP might alter brain activity [15].

Although it has long been thought that cognitive function is associated with musculoskeletal disease [16,17], whether cognitive function and posture have a direct impact on each other or to what extent they affect each other is largely unknown. Recently, the relationship between posture subluxation and cognitive function has emerged as an area of research, and evidence suggests that these two variables may be interconnected [15]. Posture subluxation, which refers to the misalignment of vertebrae in the spine relative to either a defined ideal posture or a referenced range of normal values [18], can lead to altered sensory input and proprioception [11,13], affecting the body’s postural control. This disruption in postural stability has been found to influence cognitive function [15,19]. Studies have shown that individuals with posture subluxations may experience deficits in attention, processing speed, and memory; this is especially true in older-aged individuals [20]. Moreover, aberrant proprioceptive signals resulting from subluxations can impact the brain’s ability to integrate sensory information effectively [21], where sagittal imbalance (altered posture) has been found to correlate to cognitive decline in older persons [22]. Thus, posture subluxations in the sagittal plane may not only be linked to musculoskeletal health [3,4] but could also hold potential benefits for cognitive function and overall brain health [15,17,22].

Neurophysiologically, it has been found that vertebral subluxation has a close link with altered somatosensory processing particularly in the prefrontal cortex (PFC) [23]. The prefrontal cortex is known to be a key structure responsible for the performance of what is known as “executive functions” [24]. The neuroanatomical connectivity of the PFC to most parts of the cortical and subcortical brain makes it well suited for participating in a number of neural networks and carrying out cognitive control operations in different functional domains [25]. Cognitive control is a term usually associated with the healthy functioning of the PFC and related regions such as the cingulate cortex [25].

In this context, dual-task gait, a method of assessment that evaluates gait performance while simultaneously performing a simplistic cognitive task, has emerged as a promising tool for predicting cognitive decline and assessing overall cognitive function [26]. As individuals age or experience cognitive impairments, the ability to perform dual tasks becomes more challenging due to increased cognitive demands [27]. Research has shown that gait performance during dual-task conditions can serve as a predictive indicator of cognitive decline and may help identify individuals at risk of developing cognitive impairments [28]. Furthermore, dual-task gait assessments can detect subtle changes in cognition that might not be evident during single-task gait evaluations [29]. The integration of cognitive and motor tasks in the dual-task paradigm provides a more ecologically valid measure of real-world functioning, making it a valuable tool for assessing cognitive function in various populations, including older adults and those with neurological conditions [30].

Despite the high prevalence of FHP, few studies have evaluated the effect it has on gait parameters during a dual task, where only one previous investigation was identified [31]. In this previous investigation, Moustafa and colleagues identified that whiplash-injured populations had a greater dual-task cognitive cost during walking compared to a matched population of chronic neck pain and asymptomatic controls; this increase in cognitive cost was correlated to FHP magnitude and altered sensorimotor integration as measured with the N-30 potential’s amplitude [31]. Due to the scarcity of available data on this topic, the current study was undertaken to investigate the changes in gait parameters in university students and staff with FHP when a cognitive dual task is introduced during walking. In this manuscript, we investigate the hypothesis that individuals with FHP compared to those without FHP will demonstrate different responses when undergoing dual-task assessment.

## 2. Materials and Methods

### 2.1. Study Design, Participants, and Setting

The comparative design was used to evaluate the effect of cognitive dual tasks on gait parameters in participants with forward head posture compared to a group of strictly matched control participants without FHP. The study was carried out in the laboratories of the University of Sharjah and involved participants who were students and staff members of the same institution. Recruitment efforts were conducted through social media platforms. Ethical approval was received from the University of Sharjah (College of Health Sciences, University of Sharjah, UAE) (Ethical approval number: REC-21-03-11-03-S, approval date: 25 April 2022) and ensured that all participants provided informed consent in accordance with appropriate guidelines and regulations before data collection.

### 2.2. Inclusion and Exclustion Criteria

To classify an individual with FHP, the craniovertebral angle (CVA) measurement was utilized, and established cutoff values from previous publications were adhered to. Following the data provided by Yip et al. [8], FHP was defined as having a CVA of less than 50°. Therefore, participants were categorized as having FHP if their CVA measured less than 50°. On the other hand, the control group was defined as individuals with normal or no FHP, characterized by a CVA measurement greater than 55° for each participant [7,8]. Exclusion criteria for the current investigation were as follows: (i) recent fractures; (ii) BMI > 30; (iii) a history of significant injury or primary musculoskeletal surgical interventions; (iv) deformity of the spine or extremities; and (v) pregnancy or malignancy. The student participants in our study primarily came from health sciences majors, specifically from physical therapy (43 students) and nursing (35 students). Only two participants were from computer science, ensuring that the majority perspective is from health sciences. The staff category included a diverse group of individuals from various professional roles within the institution. This category comprised administrative personnel and technical support staff.

### 2.3. Study Tools and Outcome Measures

#### 2.3.1. Craniovertebral Angle (CVA)

**Figure 1:**
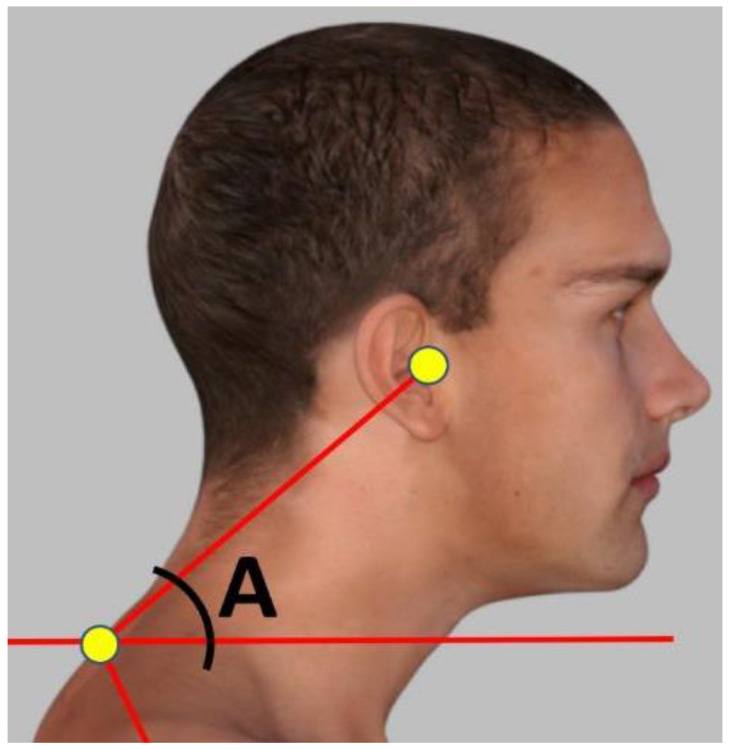
The craniovertebral angle (CVA) measured in a standing, neutral, upright posture position. The CVA (angle A) is measured by a line connecting the tragus of the ear to the C7 vertebral prominence and then measuring the intersection of this line to a horizontal line passing through C7 prominence, where the acute angle side is measured. Note: this is a computer-generated image.

The CVA is reliable and valid for the assessment of FHP [5,6,7,8,9]. To accurately measure the FHP degree, a photogrammetry method was followed measuring the CVA angle in the sagittal plane. The craniovertebral angle was measured in a standing position by using an imaginary horizontal line passing through the C7 spinous process, as well as another line from the C7 spinous process to the tragus of the ear, as shown in Figure 1. The standing posture position was used to measure the CVA as recent evidence indicates a significant difference in sitting vs. standing postures where sitting overestimates the amount of FHP (reduces the CVA) [32] and our study focused on upright gait kinematics such that the standing CVA seemed more appropriate for our analysis. The angle was determined at the point where these two lines intersected. A larger CVA value indicates a more desirable alignment of the head and neck, while a smaller angle indicates a more severe degree of FHP. Participants with an angle of less than 50° were included in the study group, while a control group was selected with a craniovertebral angle greater than 55°, matched to the study group.

#### 2.3.2. BTS GAITLAB System

**Figure 2:**
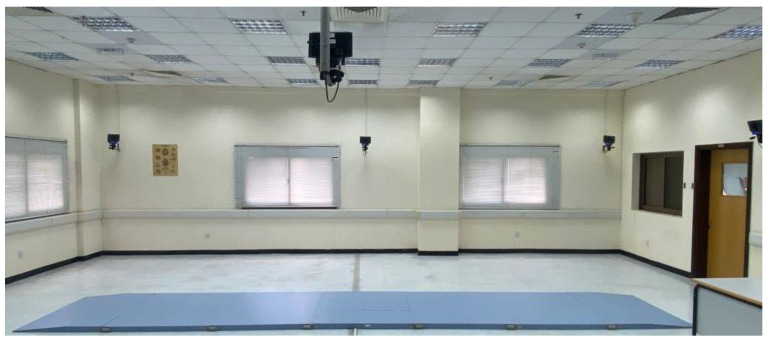

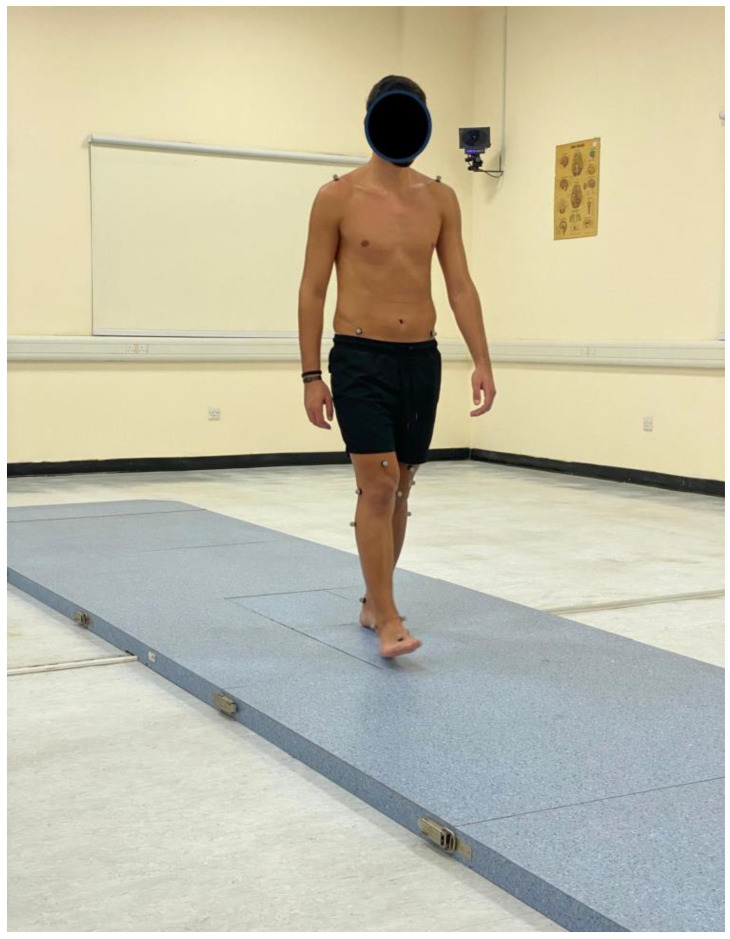
The optical motion-capture system consisting of 8 infrared cameras was used to assess gait kinematics while participants walked along the 10 m platform.

The spatiotemporal and kinematic parameters of the gait were evaluated using an optical motion-capture system consisting of 8 infrared cameras (Smart-D, BTS Bioengineering, Milan, Italy) operating at a frequency of 120 Hz (Figure 2).

**Figure 3:**
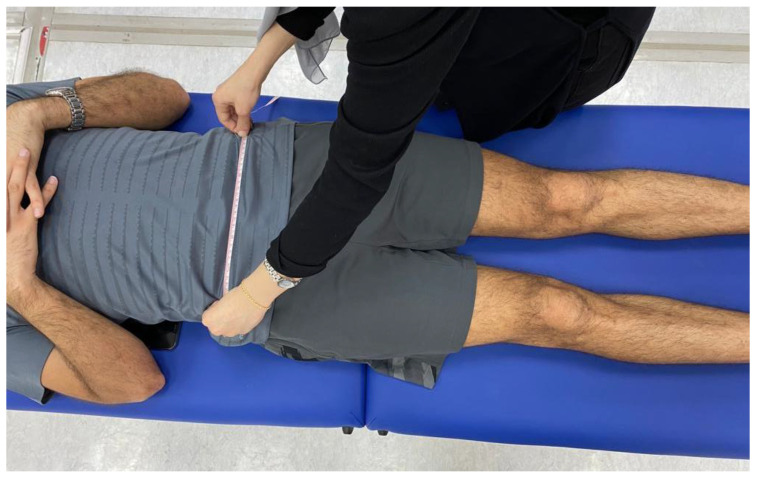
Anthropometric measurement of the distance between the anterior superior iliac spines.

**Figure 4:**
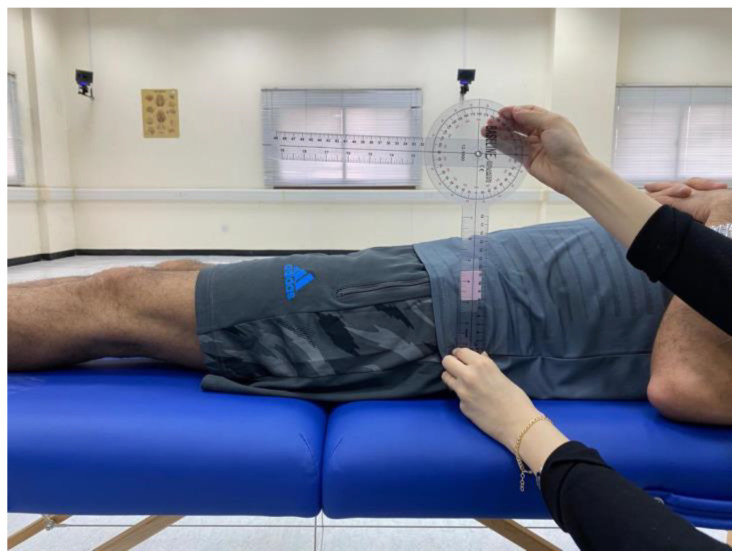
Anthropometric measurement of the pelvis thickness.

**Figure 5:**
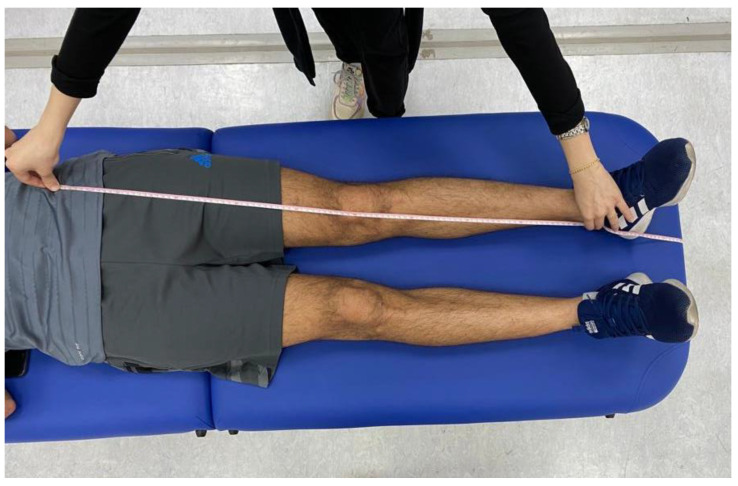
Anthropometric measurement of leg lengths was obtained bilaterally from the anterior superior iliac spine to the medial malleolus.

**Figure 6:**
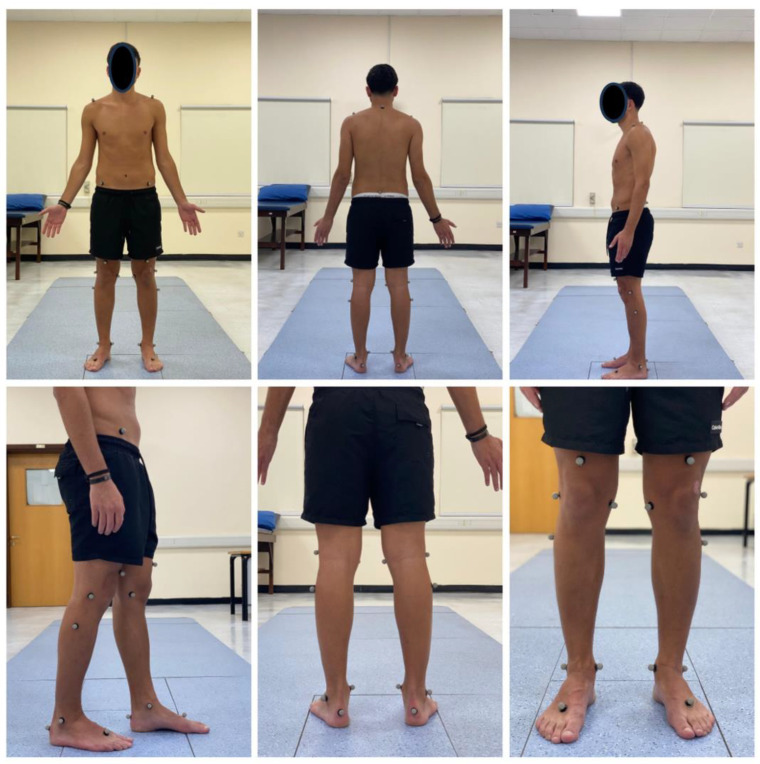
The location of the 22 spherical reflective passive markers that were placed on each participant’s skin following the protocol outlined by Davis et al. [33].

Prior to conducting the experimental tests, anthropometric measurements such as height, weight, the distance between the anterior superior iliac spines, pelvis thickness, knee and ankle width, and leg length were obtained (Figure 3, Figure 4 and Figure 5). As illustrated in Figure 6, 22 spherical reflective passive markers were placed on the participant’s skin following the protocol outlined by Davis et al. [33].

#### 2.3.3. Dual-Task Cost (DTC) Percentage

The average performance across the three dual-task trials was used for analysis to provide a robust measure of the cognitive load’s impact on gait. The dual-task cost (DTC) percentage was calculated by determining the difference between single- and dual-task performance scores, dividing the result by the single-task performance score, and then multiplying by 100. For example, if the average single-task walking speed was 1.2 m/s and the average dual-task walking speed was 1.0 m/s, the DTC percentage would be calculated as $\left(\frac{1.2-1.0}{1.2}\right)\times 100=16.67\%$.

Higher DTC percentages indicate a greater impact of the cognitive task on gait performance, highlighting the extent to which cognitive load affects the ability to maintain a normal walking pattern [34]. This method allowed for a comprehensive evaluation of the interplay between cognitive demands and gait performance in individuals with and without FHP.

#### 2.3.4. Spatiotemporal Parameters and Cognitive Performance

Participants’ spatiotemporal parameters such as step length, speed, and cadence were assessed under both single-task and dual-task conditions using the BTS gait lab system. The comprehensive gait analysis included the following 13 variables, and the left and right sides were averaged for analysis unless there was only a side-specific difference, which is then reported as side-specific in the results:

- Stride time in seconds (s);
- Stance time (s);
- Swing time (s);
- Stance phase (%);
- Swing phase (%);
- Single-support phase (%);
- Double-support phase (%);
- Mean velocity (meters (m)/s);
- Mean velocity (%height/s);
- Cadence (steps/minute (min);
- Stride length (%height);
- Step length (m);
- Step width (m).

Cognitive performance during dual-task trials was evaluated based on the accuracy and consistency of responses to cognitive tasks, providing insights into the impact of cognitive load on gait performance. The designed pathway was equipped with the BTS gait lab system to capture gait parameters in both conditions. During the dual-task trials, participants were monitored to ensure they performed both tasks simultaneously, with gentle reminders provided if necessary to maintain dual-task engagement.

### 2.4. Study Procedure

**Figure 7:**
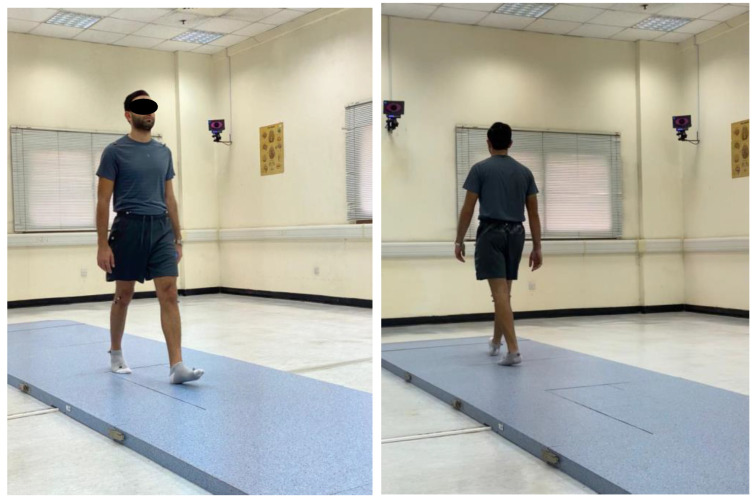
Participants were instructed to walk at their self-selected pace along a 10 m walkway while the 3D trajectories of the markers were captured by the cameras under two conditions: single-task and dual-task.

Participants were instructed to walk at their self-selected pace along a 10 m walkway while the 3D trajectories of the markers were captured by the cameras under two conditions: single-task and dual-task (Figure 7). In the single-task condition, participants walked at their preferred speed without performing any additional tasks, completing at least three trials to establish baseline gait parameters. The average performance across these trials was used for subsequent analysis [35]. For the dual-task condition, participants were instructed to walk at their preferred speed while simultaneously performing various cognitive tasks, with no emphasis on prioritizing either the gait or cognitive task. These tasks included three conditions:

(1) Answering yes or no questions;
(2) Counting forward or backward from a 3-digit number;
(3) Or performing simple mathematical calculations.

Each participant completed at least three trials for the dual-task condition, with different instructions provided for each trial. This approach ensured a comprehensive assessment of cognitive load effects [29]. A trial set was considered valid if at least 6 trials were accurately recorded, ensuring a sufficient number of gait cycles for further analysis. Rest periods between consecutive trials were allowed upon request. After completing the tests, the raw data were processed using specialized software (Smart Analyzer, BTS Bioengineering, Milan, Italy) to calculate the desired parameters.

### 2.5. Sample Size Determination

The required sample size was calculated based on the following parameters: an effect size of 0.5, representing a moderate effect size as per Cohen’s criteria; a two-tailed significance level (α) of 0.05; and a power (1 − β) of 0.80 to reduce the risk of type II errors. Using these parameters, the sample size was computed using G*Power 3.1 software. The calculations indicated that a minimum of 40 participants per group (FHP and NHP) would be needed to detect a significant difference with 80% power and a 5% level of significance. To account for potential dropouts, we added 5 participants per group, resulting in a total of 45 participants per group. Therefore, the final sample size was set at 45 participants per group, totaling 90 participants for the entire study.

### 2.6. Statistical Analysis

The distribution of all descriptive baseline variables was determined through the Kolmogorov–Smirnov test to ensure normalcy. Continuous data were presented as means accompanied by standard deviations (SDs) in both the text and tables. To evaluate equality of variances, Levene’s test was employed at a 95% confidence level, where a *p*-value < 0.05 was considered significant. Descriptive statistics (means ± SD unless otherwise indicated) were reported at each time point.

To ensure group equivalence for proper case—control analysis with each demographic and clinical variable, chi-squared tests were applied for categorical variables and Student’s *t*-tests for continuous variables. The Student’s *t*-test was used to compare the means of the continuous variables between groups, with significance set at a *p*-value < 0.05. Effect size, measured using Cohen’s d, indicated clinical importance with values of d ≈ 0.2, d ≈ 0.5, and d ≈ 0.8 representing negligible, moderate, and high clinical importance, respectively [36].

Lastly, Pearson’s correlations (r) were used to examine relationships between head posture (CVA) and variables associated with spatiotemporal gait parameters during single and dual tasks, as well as between head posture and the cognitive cost. Data analysis was performed using SPSS version 20.0 (SPSS Inc., Chicago, IL, USA), ensuring normality and equal variance assumptions were met prior to analysis.

## 3. Results

### 3.1. Demographics

**Figure 8:**
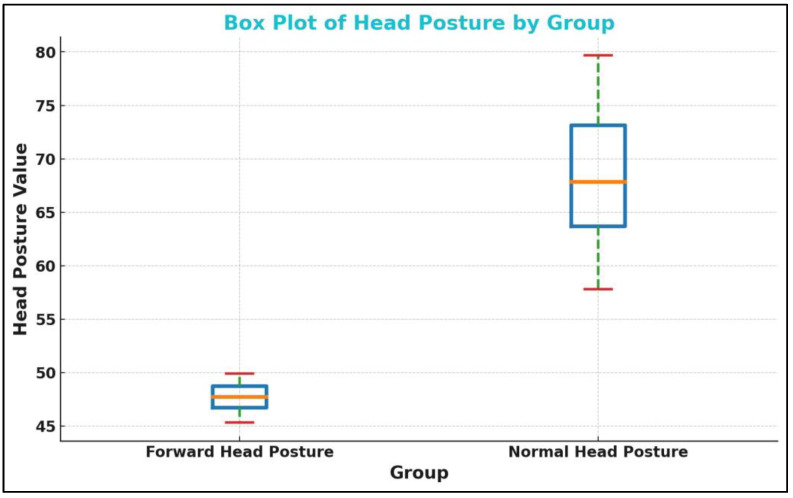
The boxplot for the craniovertebral angle (CVA) in the forward head posture (FHP) and control or normal head posture (NHP) group. The CVA is on the vertical axis shown as ‘head posture value’ (°) while the two groups are on the horizontal axis.

More than 200 potential participants were screened. Neck pain and shoulder pain were the most common reasons for participant exclusion. In total, 45 participants with FHP (25 males, 20 females) and 45 age-, BMI-, and sex-matched controls without FHP (25 males, 20 females) were recruited. See Table 1. Figure 8 shows the boxplot for the CVA.

### 3.2. Single-Task Detailed Results

In this study, several gait parameters were analyzed to compare individuals with FHP to those in a control group with neutral head posture (NHP) under single- and dual-task conditions. The mean differences between the spatiotemporal parameters in the FHP and NHP groups during single-task walking are shown in Table 2. No statistically significant differences between any of the gait variables were observed. Likewise, all the effect sizes were small to insignificant and were not statistically significant; see Table 2.

### 3.3. Dual-Task Detailed Results

The results, summarized in Table 3, showed that the stride time was significantly higher in the FHP group compared to the NHP group (*p* < 0.05), with medium to large effect sizes, indicating that participants with FHP had more difficulty maintaining the stride time. Additionally, the stance time was notably longer in the FHP group (*p* < 0.05), suggesting that these individuals might use compensatory mechanisms to maintain balance. Similarly, the swing time was significantly lower in the FHP group (*p* < 0.05), likely due to altered gait mechanics associated with their FHP.

The stance phase percentage was also significantly higher in the FHP group (*p* < 0.05), possibly reflecting a more cautious gait pattern. The double-support phase showed significant increases (*p* < 0.05), indicating a greater need for stability among those with FHP. The mean velocity was significantly lower in the FHP group (*p* < 0.05), suggesting a reduced gait speed when performing dual tasks. The stride length was shorter in the FHP group (*p* < 0.05), indicating compromised gait performance, and the step length was similarly reduced (*p* < 0.05), reflecting decreased step efficiency. Lastly, a significantly wider step width (*p* < 0.05) was observed in the FHP group, likely as a compensatory strategy for balance issues. All these findings are detailed in Table 3.

### 3.4. Cognitive Cost Detailed Results

The cognitive cost results are detailed in Table 4 and reveal significant findings across various gait parameters in the FHP group under dual-task conditions. For the stride time (both right and left), the FHP group exhibits a significantly higher cognitive cost (*p* < 0.05), indicating that dual-task conditions impose a greater cognitive load on maintaining the stride time. Similarly, the stance time (right and left) shows a higher cognitive cost (*p* < 0.05) in the FHP group, reflecting increased difficulty in managing the stance time. The swing time (right and left) also demands significantly more cognitive effort (*p* < 0.05) from the FHP group. The stance phase (right) demonstrates a higher cognitive cost (*p* < 0.05), suggesting greater challenges in maintaining this phase during dual tasks.

Additionally, the double-support phase (right) presents an increased cognitive cost (*p* < 0.05) for the FHP group, indicating difficulties in managing this phase. The mean velocity also incurs a higher cognitive cost (*p* < 0.05), signifying that maintaining walking speed is more cognitively demanding for the FHP group under dual-task conditions. The stride length (right and left) showed a significantly higher cognitive cost (*p* < 0.05), reflecting compromised gait efficiency. Similarly, the step length (right and left) incurs a higher cognitive cost (*p* < 0.05), indicating reduced step efficiency. Finally, the step width (average) reveals a significantly higher cognitive cost (*p* < 0.05) in the FHP group, suggesting a need for a broader base of support, which is cognitively demanding. Table 4 shows each of these variables and their significance.

### 3.5. Correlational Results between Variables

The correlation table for the single-task condition (Table 5) reveals the relationship between the head posture values and various performance parameters. The findings show no significant correlations, indicating that most correlations between the head posture values measured as the CVA and the performance parameters are weak and not statistically significant. This suggests that the head posture values do not have a strong influence on the performance parameters during single tasks. The lack of significant correlations indicates that head posture may not be as critical in single tasks as it is in dual tasks.

The correlation table for the dual-task testing (Table 6) illustrates a relationship between the head posture values and various performance parameters. Significant correlations include negative correlations with the stride time (s) for both the right (RT) and left (LT) sides, indicating that as the CVA decreases, both the right and left stride times tend to increase. Similarly, there are negative correlations with the stance time (s) for RT and LT, suggesting that lower CVA values are associated with shorter stance times. Additionally, there are positive correlations with the swing time (s) for RT and LT, implying that lower CVA values correlate with longer swing times.

Negative correlations with the stance phase (%) for RT and LT show that lower CVA values are associated with shorter stance phases, while positive correlations with the swing phase (%) for RT and LT indicate that lower CVA values are linked to longer swing phases. Moreover, there is a positive correlation with the step length (m) for RT and LT, demonstrating that lower CVA values correlate with longer step lengths. These correlations suggest that the CVA significantly influences the gait parameters during dual tasks, potentially affecting balance and stability.

Table 7 for the cognitive cost values highlights the relationship between the head posture values and cognitive cost measures. These correlations suggest that the head posture values significantly impact the cognitive cost associated with various gait parameters. This indicates that head posture may play a crucial role in the cognitive effort required for maintaining gait during dual tasks.

## 4. Discussion

Our study investigated the impact of FHP on cognitive dual-task performance, revealing significant findings that contribute to the existing body of knowledge on the interplay between musculoskeletal alignment and cognitive function. The results demonstrate that individuals with FHP exhibit greater cognitive dual-task costs compared to those with normal head posture, indicating that FHP may be associated with increased cognitive demands during gait performance. These results are consistent with previous studies that have demonstrated that FHP can lead to various physiological and neurological impairments, affecting both physical and cognitive functions [37]. This aligns with cognitive load theory, which suggests that increased physical discomfort and proprioceptive inaccuracies associated with FHP can elevate cognitive load, thereby impairing cognitive performance [37,38].

### 4.1. Interpretation of Findings

The observed differences in dual-task performance between the FHP and control groups align with the existing literature that suggests postural misalignments can impact cognitive processes [15,22,31,37,38]. The increased cognitive cost in the FHP group may be attributed to altered sensory input and proprioception resulting from vertebral misalignments. Similar to how phantom limb pain develops due to maladaptive cortical reorganization in response to the loss of sensory input [39], FHP can lead to maladaptive changes in the sensory and motor cortex. These disruptions potentially lead to increased cognitive load as the brain compensates for impaired postural stability, as supported by studies indicating deficits in attention, processing speed, and memory in individuals with postural deviations [22,40]. The increased cognitive cost observed in the FHP group can be attributed to several underlying mechanisms. Firstly, FHP causes biomechanical stress on the neck and shoulders, leading to muscle fatigue and discomfort, which in turn can reduce cognitive resources available for task performance [38,41]. Secondly, FHP is associated with altered proprioceptive function, which can disrupt sensory input and integration, further increasing the cognitive load required for maintaining balance and performing tasks [11,13,14,41]. Finally, the structural changes in the cervical spine due to FHP, through alteration in the cervical lordosis, can impair blood flow to the brain [42], affecting cognitive functions such as attention and memory [22,37].

Additionally, deafferentation resulting from faulty posture may very well exacerbate cognitive impairments. Research has shown that the loss of sensory nerve signals due to poor posture may increase cognitive load and impair cognitive performance by disrupting proprioception feedback and increasing the brain’s compensatory efforts [22,31,43]. Moreover, neuroplasticity plays a critical role in the relationship between cognition and posture. Neuroplastic changes in response to altered sensory input from poor posture can lead to both adaptive and maladaptive cognitive outcomes. Studies indicate that neuroplasticity mechanisms, such as synaptogenesis and cortical reorganization, are involved in adapting to increased cognitive demands [44,45] and it is possible that these same adaptations are imposed by FHP. These changes highlight the brain’s ability to reorganize and modify its neural connections in response to environmental stimuli and experience, which is crucial for maintaining cognitive function in the face of physical impairments [45].

### 4.2. Implications for Clinical Practice

The significant correlations between the CVA and cognitive dual-task performance emphasize the need for comprehensive assessment protocols that consider both musculoskeletal and cognitive parameters. Clinicians should be aware of the potential cognitive implications of FHP and incorporate strategies to address postural alignment in rehabilitation programs. This approach is particularly relevant for populations at risk of cognitive decline, such as older adults and individuals with neurological conditions [22].

Our study also supports the use of dual-task gait assessments as a valuable tool for detecting cognitive impairments. The integration of cognitive and motor tasks in these assessments provides a more ecologically valid measure of real-world functioning, which is critical for identifying subtle cognitive deficits that may not be evident in single-task evaluations [46,47]. This has significant implications for early intervention and monitoring of cognitive health in clinical settings. Clinicians should consider assessing posture as part of routine evaluations, especially for individuals presenting with cognitive complaints. Interdisciplinary approaches involving physical therapists, occupational therapists, and cognitive specialists can provide comprehensive care that addresses both the physical and cognitive challenges associated with FHP. Early intervention and education on the importance of maintaining proper posture could help prevent the development of FHP and, therefore, any associated cognitive impairments [34,35].

### 4.3. Practical Applications

Individuals with FHP are likely to experience increased cognitive fatigue and reduced efficiency in tasks requiring simultaneous cognitive and physical effort. This could impact daily activities, workplace productivity, and overall quality of life. For example, Jung and colleagues recently identified that office workers who adopted a forward head posture had a change in the magnitude and frequency of gamma brain waves [15]. Specifically, it was found that reducing the CVA (increasing FHP) caused brain waves to switch to gamma waves at rest. Higher gamma waves are important during cognition and mental processing and are linked to higher IQs; however, at rest, high gamma activity is not normal and is associated with greater sympathetic demand [15]. Jung et al. [15] stated “*the activity of gamma waves during rest is related to the abnormal excitatory system and hyperarousal of the sensory system and can affect the overall level of neural excitation, causing unnecessary arousal and interfering with psychological relaxation*”. Furthermore, chronic FHP can lead to long-term health issues, including chronic neck pain, headaches [4,5], and potential cognitive decline [22,34,35]. To mitigate the negative effects of FHP, interventions aimed at correcting posture should be considered. These could include physical therapy, ergonomic adjustments in the workplace, and exercises designed to strengthen the cervical and upper back muscles. Incorporating regular breaks and posture checks during prolonged computer use can also help in maintaining proper alignment and reducing the risk of developing FHP [4,15].

### 4.4. Limitations and Future Directions

Despite the robust design and significant findings of our study, several limitations must be acknowledged. The cross-sectional nature of the study precludes causal inferences, and longitudinal studies are necessary to establish the temporal relationship between FHP and cognitive decline. Additionally, our sample was limited to university students and staff, which may not be representative of the general population; in fact, an older cohort known to have cognitive decline may show greater gait alterations with postural misalignment with a dual-task cognitive challenge. Future research should include diverse populations to enhance the generalizability of the findings. Further investigation into the mechanisms underlying the relationship between posture and cognitive function is warranted. Specifically, exploring the role of different regions of the brain and their connectivity in individuals with postural deviations can provide deeper insights into the neurophysiological basis of our findings. Additionally, interventional studies examining the effects of postural correction on cognitive outcomes will be valuable in determining the efficacy of targeted therapies.

## 5. Conclusions

Our study demonstrates that forward head posture significantly increases the cognitive cost during walking, highlighting the importance of proper postural alignment for maintaining cognitive function. Addressing FHP through targeted interventions could improve both physical and cognitive health, enhancing overall well-being and daily functioning. Further research is needed to explore the long-term effects of FHP and the efficacy of different interventions in mitigating its cognitive impacts.

## Figures and Tables

**Table 1:** Baseline demographic characteristics and clinical variables. FHP: forward head posture group; NHP: normal head posture group; BMI: body mass index; CVA: craniovertebral angle.

Variable		FHP (n = 45)	NHP (n = 45)
Age (years)		20.5 ± 2	20 ± 3
BMI (kg/m^2^)		21.5 ± 1.7	21.4 ± 1.8
**Gender (%)**			
Male		25 (55.5%)	25 (55.5%)
Female		20 (44.5%)	20 (44.5%)
CVA (°)		46.64 ± 3.23	67.95 ± 7.89
**Participants categorization**			
Health Sciences Students	Physical Therapy	23 (25.5%)	20 (22.2%)
	Nursing	16 (17.8%)	19 (21.1%)
Computer Science Students		-	2 (2.2%)
Staff Members (Administrative, Technical)		6 (6.7%)	4 (4.5%)

**Table 2:** Mean differences between spatiotemporal parameters in forward head posture (FHP) and neutral head posture (NHP) groups during single-task walking are shown.

Variable	Group	Mean ± SD	95% CI	Effect Size (d)	*p*-Value
Stride time	FHP	1.12 ± 0.08	[−0.061, 0.041]	−0.11	0.60
	NHP	1.13 ± 0.12			
Stance time	FHP	0.67 ± 0.07	[−0.074, 0.014]	−0.29	0.30
	NHP	0.70 ± 0.11			
Swing time	FHP	0.43 ± 0.05	[−0.025, 0.025]	0.06	0.30
	NHP	0.43 ± 0.05			
Stance phase	FHP	60.19 ± 3.99	[−2.59, 1.69]	−0.11	0.20
	NHP	60.64 ± 4.12			
Double-support phase	FHP	10.81 ± 2.19	[−1.60, 0.54]	−0.25	0.20
	NHP	11.34 ± 2.07			
Mean velocity	FHP	1.16 ± 0.13	[−0.11, 0.054]	−0.21	0.15
	NHP	1.19 ± 0.17			
Mean velocity height	FHP	70.62 ± 8.29	[−5.90, 3.24]	−0.13	0.26
	NHP	71.95 ± 10.91			
Cadence	FHP	108.12 ± 7.91	[−4.25, 5.79]	0.08	0.60
	NHP	107.35 ± 9.93			
Stride length	FHP	78.46 ± 7.50	[−5.27, 2.31]	−0.19	0.40
	NHP	79.94 ± 7.51			
Step length	FHP	0.64 ± 0.06	[−0.036, 0.036]	0.13	0.80
	NHP	0.64 ± 0.08			
Step width average	FHP	0.08 ± 0.03	[−0.047, 0.027]	−0.24	0.90
	NHP	0.09 ± 0.06			
Swing phase (%)	FHP	38.91 ± 2.83	[−1.51, 0.39]	0.20	0.40
	NHP	38.35 ± 2.74			
Single-support phase (%)	FHP	39.45 ± 2.52	[−0.62, 1.075]	−0.08	0.72
	NHP	39.67 ± 2.77			

**Table 3:** Mean differences between spatiotemporal parameters in forward head posture (FHP) and neutral head posture (NHP) groups during dual task.

Variable	Group	Mean ± SD	95% CI	Effect Size (d)	*p*-Value
**Stride time**	FHP	1.40 ± 0.32	[0.118, 0.322]	0.88	<0.001
	NHP	1.18 ± 0.14			
**Stance time**	FHP	0.72 ± 0.06	[0.051, 0.109]	1.14	<0.001
	NHP	0.64 ± 0.08			
**Swing time**	FHP	0.44 ± 0.04	[−0.074, −0.026]	−0.95	<0.001
	NHP	0.49 ± 0.06			
**Stance phase**	FHP	65.01 ± 5.97	[2.044, 5.656]	0.83	0.01
	NHP	61.16 ± 2.33			
**Double-support phase**	FHP	12.29 ± 1.41	[1.232, 1.628]	0.94	<0.001
	NHP	10.86 ± 1.61			
**Mean velocity**	FHP	1.08 ± 0.12	[−0.304, −0.116]	−0.96	<0.001
	NHP	1.29 ± 0.30			
**Mean velocity height**	FHP	60.86 ± 7.92	[−14.45, −7.25]	−1.25	<0.001
	NHP	71.71 ± 9.46			
**Cadence**	FHP	103.18 ± 10.13	[−5.29, 2.99]	−0.18	0.30
	NHP	104.83 ± 7.56			
**Stride length**	FHP	73.08 ± 7.37	[−11.03, −4.57]	−1.00	<0.001
	NHP	80.88 ± 8.19			
**Step length**	FHP	0.58 ± 0.06	[−0.105, −0.035]	−1.10	<0.001
	NHP	0.65 ± 0.08			
**Step width average**	FHP	0.15 ± 0.23	[0.013, 0.147]	0.48	<0.001
	NHP	0.07 ± 0.02			
**Swing phase (%)**	FHP	38.36 ± 1.83	[0.88, 3.37]	−0.90	0.0008
	NHP	40.69 ± 3.17			
**Single-support phase (%)**	FHP	39.37 ± 2.16	[0.68, 4.35]	−0.65	0.01
	NHP	41.96 ± 5.18			

**Table 4:** The mean difference in cognitive cost (CC) between forward head posture (FHP) and normal head posture (NHP). CC is calculated as the difference in parameter values from single to dual task divided by the single-task value, multiplied by 100%.

Variable	Group	Mean ± SD	95% CI	Effect Size (d)	*p*-Value
CC Stride time	FHP	25.79 ± 29.76	[10.90, 30.30]	0.87	<0.001
	NHP	5.19 ± 14.72			
CC Stance time	FHP	8.43 ± 19.25	[8.20, 22.16]	0.89	<0.001
	NHP	−6.75 ± 14.09			
CC Swing time	FHP	2.78 ± 10.64	[−19.34, −5.16}	−0.72	<0.001
	NHP	15.03 ± 21.86			
CC Stance phase	FHP	8.49 ± 12.79	[2.56, 11.62]	0.64	0.01
	NHP	1.40 ± 8.74			
CC Double-support phase	FHP	19.87 ± 37.99	[5.85, 31.67]	0.66	0.01
	NHP	1.11 ± 22.62			
CC Mean velocity	FHP	12.36 ± 34.86	[−5.20, 16.22]	−0.75	0.02
	NHP	6.85 ± 11.41			
CC Mean velocity height	FHP	13.34 ± 13.09	[1.60, 18.72]	−0.80	<0.001
	NHP	3.18 ± 26.27			
CC Cadence	FHP	4.49 ± 8.01	[−1.16, 7.22]	−0.30	<0.001
	NHP	1.46 ± 11.93			
CC Stride length	FHP	6.84 ± 8.54	[−0.35, 9.21]	−0.81	0.01
	NHP	2.41 ± 14.00			
CC Step length	FHP	9.98 ± 10.65	[0.33, 10.85]	−1.13	0.01
	NHP	4.39 ± 14.51			
CC Step width average	FHP	12.43 ± 340.60	[0.31, 4.73]	0.49	0.01
	NHP	9.91 ± 40.58			
Swing phase (%)	FHP	15.79 ± 3.7	[6.51, 10.87]	2.03	<0.001
	NHP	7.1 ± 4.8			
Single-support phase (%)	FHP	17.36 ± 1.86	[6.22, 10.90]	1.51	<0.001
	NHP	8.8 ± 7.8			

**Table 5:** Correlation between the craniovertebral angle (CVA) and gait parameters during single-task conditions. s: seconds; m: meters.

Parameter	Pearson’s r	*p*-Value
Stride time (s)	0.02	0.86
Stance time (s)	0.08	0.52
Swing time (s)	0.1	0.98
Stance phase (%)	0.04	0.77
Swing phase (%)	−0.03	0.98
Single-support phase (%)	0.06	0.61
Double-support phase (%)	0.12	0.29
Mean velocity (m/s)	0.08	0.49
Mean velocity (%height/s)	0.05	0.67
Cadence (steps/min)	−0.02	0.90
Stride length (%height)	0.05	0.60
Step length (m)	−0.03	0.70
Step width (m)	0.03	0.80

**Table 6:** Correlation between the craniovertebral angle (CVA) and gait parameters during dual task. s: seconds; m: meters.

Parameter	Pearson’s r	*p*-Value
Stride time (s)	−0.68	<0.001
Stance time (s)	−0.56	<0.001
Swing time (s)	0.31	<0.001
Stance phase (%)	−0.56	<0.001
Swing phase (%)	0.53	<0.001
Single-support phase (%)	0.40	<0.001
Double-support phase (%)	−0.55	<0.001
Mean velocity (m/s)	0.28	<0.001
Mean velocity (%height/s)	0.49	<0.001
Cadence (steps/min)	0.20	<0.001
Stride length (%height)	0.39	<0.001
Step length (m)	0.60	<0.001
Step width (m)	−0.40	<0.001

**Table 7:** Correlation between the craniovertebral angle (CVA) and cognitive cost (CC) per each parameter. s: seconds; m: meters.

Parameter	Pearson’s r	*p*-Value
CC Stride time (s)	−0.61	<0.001
CC Stance time (s)	−0.41	<0.001
CC Swing time (s)	−0.35	<0.001
CC Stance phase (%)	−0.35	<0.001
CC Swing phase (%)	−0.45	<0.001
CC Single-support phase (%)	−0.23	0.04
CC Double-support phase (%)	−0.21	0.04
CC Mean velocity (m/s)	−0.37	0.01
CC Mean velocity (%height/s)	−0.30	0.01
CC Cadence (steps/min)	−0.22	0.01
CC Stride length (%height)	−0.32	0.01
CC Step length (m)	−0.55	<0.001
CC Step width (m)	−0.50	<0.001

## Data Availability

The datasets analyzed in the current study are available from the corresponding author on reasonable request.
